# New technologies applied to canine limb prostheses: A review

**DOI:** 10.14202/vetworld.2021.2793-2802

**Published:** 2021-10-28

**Authors:** Paul G. Arauz, Patricio Chiriboga, María-Gabriela García, Imin Kao, Eduardo A. Díaz

**Affiliations:** 1 Department of Mechanical Engineering, Universidad San Francisco de Quito, Quito, Ecuador; 2 Department of Industrial Engineering, Universidad San Francisco de Quito, Quito, Ecuador; 3 Department of Mechanical Engineering, Stony Brook University, Stony Brook, United States; 4 Department of Veterinary Medicine, Universidad San Francisco de Quito, Quito, Ecuador.

**Keywords:** canine exo-prosthesis, canine endo-exo prosthesis, canine prosthetics, Osseointegration, canine limb biomechanics

## Abstract

Although only a few studies have investigated about the development of animal prosthesis, currently, there is an increasing interest in canine limb prosthesis design and its clinical application since they offer an alternative to killing the animal in extreme situations where amputating the limb is the only option. Restoring normal function of amputated canine limbs with the use of a prosthesis is challenging. However, recent advances in surgical procedures and prosthesis design technology appear promising in developing devices that closely recreate normal canine limb function. Surgical advances such as evolution of osseointegration (bone-anchored) prostheses present great promise. Likewise, modern computer-aided design and manufacturing technology, as well as novel motion analysis systems are now providing improved prosthesis designs. Advances in patient-customized prostheses have the potential to reduce the risk of implant failure. The objective of this investigation is to present a general review of the existing literature on modern surgical approaches, design and manufacturing methods, as well as biomechanical analyses so that veterinarians can make more and better-informed decisions on the development and selection of proper canine limb prosthesis. Isolated research efforts have made possible an improvement in stability, comfort, and performance of canine limb prosthesis. However, continued multidisciplinary research collaboration and teamwork among veterinarians, engineers, designers, and industry, with supporting scientific evidence, is required to better understand the development of canine limb prosthesis designs that closely replicate the normal limb function.

## Introduction

Throughout the world, only a few studies have investigated dog ownership predictors [1-7]. For instance, according to the American Veterinary Medical Association [1], 60% and 46% of the United States’ population own at least one pet and a dog, respectively. The interaction between dogs and humans is physically and emotionally beneficial for both species [8,9]. All this has led to the advancement of veterinary medicine and treatment, based on the introduction of innovative technologies and surgical improvements in such areas [10]. In particular, several companies [11-13] are now providing hundreds of customized canine prosthetics. However, there is limited scientific evidence supporting the development and efficacy of canine limb prosthesis, with only few research groups reporting on a case-by-case basis [14-18].

Among the most common indications for amputating canine full limbs are: Neoplasia with irreversible neurologic compromise, severe trauma, ischemic necrosis, uncontrollable orthopedic infections, paralysis, unmanageable arthritis, congenital deformity, and in most cases osteosarcoma [19]. For example, osteosarcoma, the most prevalent canine primary tumor, affects over ten-thousand dogs each year in the United States, and the amputation of limbs is widely accepted as the preferred treatment for localized primary bone and joint tumors in canines [19-21]. Consequently, there is an emerging interest in canine limb prosthesis design and its clinical application since they offer an alternative to killing the canine in extreme situations where amputating the limb is the only option [21-23]. Modern veterinary medicine now includes not only sophisticated procedures such as joint replacements, stereotactic radiation, chemotherapy, and advanced dentistry but also the application of biomechanics and modern technologies when treating limb loss and/or loss of limb function [24,25].

Cutting-edge prosthetic innovation creates a conundrum when attempting to select what the best prosthetic is for a specific canine patient. Therefore, the goal of this investigation was to present a general review of the existing literature on modern surgical approaches, design and manufacturing methods, and biomechanical analyses so that veterinarians can make more and better-informed decisions on the development and selection of proper canine limb prosthesis.

## Prosthesis Development

The need to recover the function of lost limbs generated the development of human prostheses. In fact, historical proof indicates that prostheses were being used as early as the fifteenth century BC [26]. Among its collection, the Cairo Museum exhibits a mummy showing amputation of its right great toe and replacement with a prosthesis made of leather and wood [26,27]. The fact that many soldiers lost limbs during wars such as the American Civil War, World War I, and World War II produced important surgical amputation advances and prosthesis refinements [26]. Veterinary medicine has adapted human technologies and surgical procedures in the development of animal prostheses. In fact, recently, several studies have reported on the successful replacement of lost and/or damaged limbs, beaks, fins, and tails with man-made devices [28-30]. This is a clear example of the adaptation from human to veterinary medicine enclosing treatment, surgical, and rehabilitation procedures, which has dramatically changed how diseased animals are treated in the veterinary setting [31].

The appropriate materials, design, construction, and alignment must be considered in the development of a prosthesis to satisfy the functional needs of the user. In general, the objectives of canine limb prostheses are: Imparting an improved quality of life, preventing further deformation and degeneration of existing joints, reducing limb length discrepancies, raising exercise and activity levels, offering a way to take part in rehabilitation therapy, and being capable of executing daily life activities [16,24,25]. Among the required aspects for a prosthesis to be functionally consistent are body support, shock absorption, energy storage and return, and function flexibility; and if pertinent surgical planning, precise design, manufacturing, and testing of the prosthesis, or adequate prosthesis placement are not considered, problems may appear [16,24,25]. Furthermore, introduction of biomechanical aspects, not formerly included in veterinary medicine and the prosthesis industry, such as coupling forces, comprehension of quadrupedal locomotion and gait, as well as biomechanical research and analysis of veterinary patients, are required for the use of prostheses [24,25].

**Figure-1:**
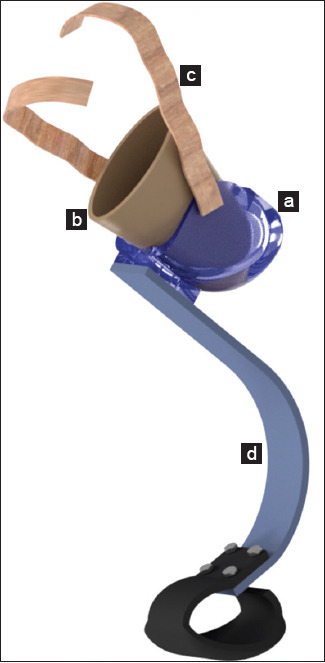
A schematic of a hind-limb canine exo-prosthesis indicating: (a) socket; (b) liner; (c) belt suspension system; (d) shock-absorbing pylon.

**Figure-2:**
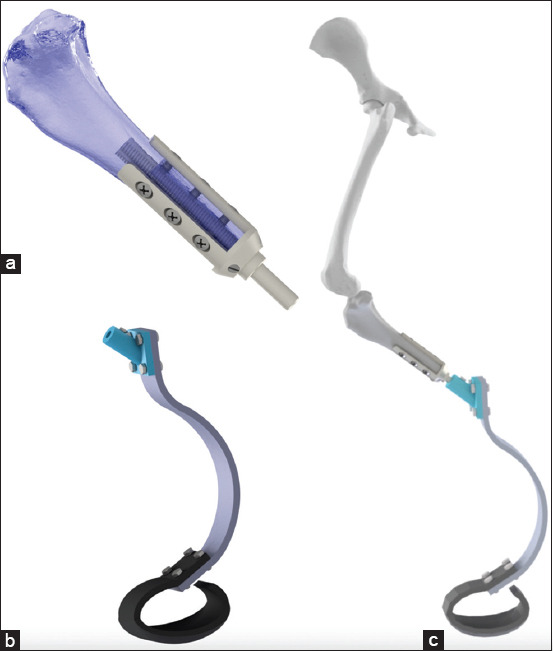
(a) A schematic of an osseointegrated transtibial hind-limb canine endo-prosthesis intramedullary implanted; (b) a schematic of an exo-prosthesis to be attached to the endo-prosthesis; (c) a schematic of the assembled hind-limb endo-exo-prosthesis.

Nowadays, canine limb prosthesis can be classified in two types: (1) Exo-prosthesis and (2) endo-exo prosthesis. Despite the lack of enough published scientific evidence, conventional exo-prosthesis using external sockets and suspension systems are mostly prevalent [11,12,14-16,32] (Figure-1). Conversely, endo-prosthesis, which incorporates the prosthesis into the remaining bone through osseointegration, is also an option, but its implementation in veterinary medicine is not very common [10,18,33] (Figure-2).

A canine limb exo-prosthesis typically consists of four elements, such as a socket, a liner, a suspension system, and a shock-absorbing pylon (Figure-1).

In general, the prosthetic socket is the principal element of a prosthesis. Similar to human prostheses, socket design in the canine limb-prosthesis interface should properly provide stability, acceptable load transmission, comfort, and effective mobility control [34-37]. The purpose of a socket is not just to accommodate forces or loads propagating across the residual limb comfortably. For instance, a reasonable assumption is that as a canine with a missing limb walks, the residuum muscles first develop a compensatory contraction strategy to create a closed kinematic chain within the socket for structural stability, and then continuous sequential contractions are generated to control the prosthesis during functional movement. In socket design, detailed attention to residuum soft tissues tolerance to pressure variations and repetitive forces encountered when wearing a prosthesis is needed. Indeed, proper knowledge and comprehension of the residuum anatomy and soft-tissue biomechanics lead to socket designs transferring forces from the prosthesis to the residuum more efficiently without damaging the soft tissue or skin [26,35,37]. In humans, proportional decreases between positive pressure and skin irritation, as well as tissue breakdown have been reported [38,39]. It has been stated that the higher the negative pressure, the better the circulation within the residuum, causing better nutrition and health to the tissues, or a faster healing process [38]. At present, commercially available canine limb suction socket designs made of a gel suspension liner [11,13], exist. However, because little scientific evidence in humans and no evidence at all in dogs exist, a close follow-up of patients with these socket designs is necessary to avoid potential adverse effects from prolonged use or the relationship between negative-pressure sockets and the vascularity in the residuum [26].

As indicated by its name, a suspension system suspends or sustains the socket in position. The function of a liner is to pad the residuum with comfortable soft material. In practice, liners and suspension devices are typically integrated to accommodate their corresponding functions using several materials and combinations such as foam, rubber, silicone, silico gels, elastomers, urethanes, elastic polymers, and neoprene, among others. Although there are no studies reporting on suspension systems for canine limb prosthesis, common advantages reported with the use of similar materials, such as silicone liners, in human limb prosthesis include reduced skin irritation and pain, as well as better comfort and socket-residuum fitting [40-42]. A liner with a reduced compressive stiffness may provide better padding and socket-residuum fitting in a dog with a slim or thin residuum. In contrast, a liner with an increased compressive stiffness may improve prosthetic control in a dog with a corpulent residuum. Although directly quantifying these measures is difficult, computational modeling and numerical analysis are an alternative that can help veterinarians to estimate such measures. At present, suspension systems commercially available are: (1) Self-suspension of the socket, utilizing the residuum anatomical geometry, and in some cases compromising the knee joint; (2) suction suspension, consisting of a suction socket design made of a gel suspension liner; and (3) suspension harnesses, including belts, sleeves, cuffs, straps, and wedges [11-13].

A shock-absorbing pylon is a prosthetic element intended to reduce the shock forces generated during the execution of distinct high-impact activities. These devices are spring-like mechanisms that are fitted according to the level of limb amputation. For instance, in a dog having a transtibial amputation, the shock-absorbing pylon is typically fitted in the tibial and paw sections of the prosthesis to reduce the impact forces associated with walking [14,15]. However, limited studies reported objective scientific evidence on canines fitted with shock-absorbing pylons [14,15]. In humans, pylon primarily consists of metals such as stainless steel, titanium, and aluminum [42]. The same metals may be used in canine limb prosthesis design, yet the decision on which particular metal to use will need to be made on an individual basis. For example, for a small dog, a pylon made of aluminum may be more appropriate due to its light weight and reduced cost in comparison to stainless steel and titanium, respectively.

## Osseointegration

Osseointegration is a direct structural and functional connection between bone and a metallic implant (the stable integration of metal implants into bone), and it has become a positive surgical innovation to circumvent many of the complications and restrictions inherent to socket-based prostheses. Osseointegration was originally investigated in humans [43-45], and it is intended to ameliorate functionality, durability, and freedom of motion in the prosthesis as well vibrotactile and pressure feedback secondary to osseoperception [43-46]. However, despite the good short-term results reported in several studies, the technique still remains controversial [47-50]. Among the main concerns are the mechanical longevity of the implant system, durability of the bone anchorage, and rate of infection [48]. Similar to humans, the challenges of canine exo-prosthetic use are to overcome issues related to socket prostheses. Several socket-related issues include chafing, pain, skin sores, and perspiration [48]. Osseointegration typically incorporates titanium implants into the bone intramedullary canal (Figure-2). A percutaneous fixture is inserted through the skin, into the intramedullary component and secured with the percutaneous fixture to which the exo-prosthesis is attached. This technique eliminates direct skin-socket contact producing an anchor for the prosthetic limb without compromising the skin or soft tissues [26,46] (Figure-2).

Two osseointegration systems have been examined in canine studies. One of them, the United States produced Alameda East Veterinary Hospital BioMedtrix (AEVHBM) has indicated an outcome for more than 1 year for a load-bearing prosthesis [51]. The AEVHBM implant system was uniquely designed for single surgery implantation of a single-component system into both pelvic limbs of a canine [51]. After 14 months, complications due to failed osseointegration caused the removal of one of the systems. Yet, implantation of a newly designed system indicated no complications with a 26-month follow-up posterior to the initial surgery. The failed system included a threaded tapered intramedullary stem made of an alloy of Ti6Al4 V. A coating of porous tantalum at the distal end of the stem facilitated soft tissue integration and acted as a collar towards the distal end of the bone. In the newly designed system, an unthreaded stem with longitudinal splines was utilized instead of the tapered thread to reduce rotational instability [51] (Table-1).

**Table 1:** Osseointegration canine limb prosthesis studies: system, interface characteristics, material, manufacturer, number of patients, and outcomes.

Study	Prosthesis system	Material	Bone- implant	Percutaneous part – soft tissue	Manufacturer	Patients	Outcome
Drygas *et al*. [51]	Alameda East Veterinary Hospital BioMedtrix (AEVHBM)	Ti6Al4 V alloy	Thread/press-fit	Porous tantalum	Custom-made Alameda East Veterinary Hospital, Denver CO, US	1-dog	14-26 months survival
Fitzpatrick *et al*. [19]	Intraosseous transcutaneous amputation prosthesis (ITAP)	Ti6Al4 V alloy	Press-fit	Polished	Custom-made Institute of Orthopaedics of University College, London, UK	4-dogs	12* months survival

* Outcome based on one dog only because other dogs had to be euthanized [19]. Thus, such outcome may have been reduced due to problems unrelated to the prostheses.

The other system is the Intraosseous Transcutaneous Amputation Prosthesis (ITAP). Fitzpatrick *et al*. [19] reported on the clinical application of ITAP for canines with limb amputation. In one canine, a successful ITAP replacement was achieved following an ITAP fracture that occurred 10 weeks after the initial surgery. Even though confirmation or assumption of metastatic disease at 8, 12, and 17 months led to euthanize three canines, osseous, and dermal integration was indicated by a histologic examination of the ITAP limb interface at 1 year [19]. Although the success of the implants may have been reduced due to problems unrelated to the prostheses, the authors concluded that feasibility and favorable functional outcomes can be expected by implanting the distal limb of a canine with an ITAP system. It was also claimed that a reliable and robust biological integration of osseous and dermal tissues was possible with an ITAP system [19] (Table-1).

Future developments of bone-anchored prosthesis may address several aspects, such as mechanical longevity of the implant system [48], durability of the bone anchorage [48], rate of infection [48], lengthy recovery time before loading [19], the incidence of failed osseointegration [51], mechanical failures in high-impact activities [19], the use of adequate materials to guarantee biocompatibility avoiding infection, and similar to advances in human osseointegration the alternative to offer closed-loop, neuro-muscular control of the limb prosthetics [46].

## Computer Design

Biomechanical design is required to improve canine limb prosthesis function. Computer-aided drawing and three-dimensional (3D) designing systems are raising the level of accuracy of prosthesis design into high function and mechanical precision [25]. In part, this is possible due to novel tools and technologies applied to capture residual limb and/or bone shapes such as 3D-scanning, computed tomography (CT) scan, or magnetic resonance imaging (MRI). As a case in point, software can be used to render a 3D solid model from the 3D-scanning, CT scan, or MRI of limbs and joints, which can be brought to inform the design of prosthesis limb functions. In general, computer-aided design tools adopt Digital Imaging and Computing in Medicine standard for acquiring, processing, and storing medical images and emits volumetric outputs of the designed parts in stereolithography and object standard formats that are compatible with 3D-printers [10,18,20].

Once 3D prosthesis design models are completed, computational finite element simulations are applied to assess the performance of the prosthesis under real-world force and stress conditions, as well as the prosthesis biomechanical function [52]. In fact, several studies have developed finite element methods inspired by the biomechanical test setups in humans [14,18,32,53-55].

### 3D-scanning

**Figure-3:**
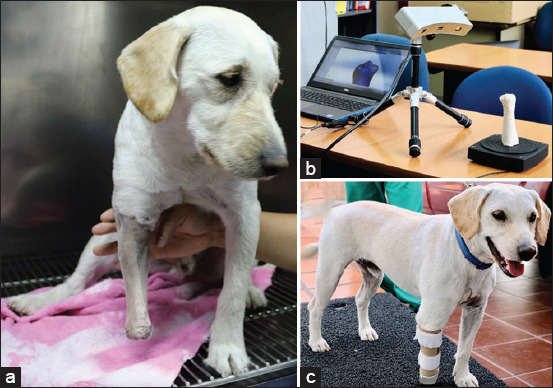
(a) A photo displaying a dog with its right-front residual limb prepared to be casted; (b) A photo showing a three-dimensional (3D)-scanning of a casted residual limb of the dog; (c) A photo presenting the dog with a front-limb exo-prosthesis constructed by 3D-scanning and computer design approaches.

A common exo-prosthesis design approach utilizes 3D scanners that capture limb topography directly from the patient’s residual limb [56] or from a cast of the residual limb for digital sculpting and computer modeling [57] (Figure-3). For instance, Kastlunger [14] designed and developed a custom prosthetic for a canine born with a congenital right forelimb deformity. The cast of the canine’s thorax was 3D scanned from a 360-degree angle view using an Xbox 360 Kinect to produce a custom-made prosthetics with a satisfactory socket that was accurately adjusted to the canine’s body segment. The author reported that the prosthesis built (3D-printed and including a pylon structure) presented sufficient strength to resist high-impact forces and stresses generated during the canine gait.

Currently, several companies [11-13] offer commercially available custom-made 3D-printed prosthetics using 3D scanners that capture limb topography directly or from a cast. The costs of commercially available prosthetics may range approximately between $1,000 and $2,000 depending on size and specific features on each device, and it is claimed that these prosthetics are lightweight, waterproof, breathable, and flexible in the areas needed [11-13].

### CT

Recent canine studies [10,18,20] presented the development of customized implants utilizing a CT scan to reconstruct bone structures and limb of the patient, and these implants being fabricated by applying metal additive manufacturing technologies. For instance, Séguin *et al*. [20] used CT scans of the thoracic limbs to create patient-specific endoprostheses and cutting guides for limb-sparing in dogs. The authors reported a good to excellent fit between host bones with the cutting-guide and endoprosthesis. It is well known that a CT scan uses a sophisticated technology that can precisely reproduce bone dimensions. However, digital and physical discrepancies in bone dimensions are possible due to distinct sources of error such as scanning parameters, scanner reconstruction algorithms, surface reconstruction parameters, as well as, printing resolution, landmark selection, and ruler positioning, among others [58,59]. Therefore, proper considerations are required to produce accurate 3D models of canine limbs using this technique.

### MRI

Similar to a 3D-scanner, an MRI can be utilized to develop 3D limb and bone model structures. Although no studies have reported on the use of MRI for canine limb prothesis modeling, MRI is frequently used as a diagnostic and assessment tool of internal limb structures [60,61]. For example, a recent study reported on the utility of MRI as a diagnostic tool for evaluating tendon pathology [60]. The authors concluded that an extraordinary level of detail of the tendons that comprise the common calcaneal tendon and the anatomical relationships to surrounding structures were possible with the use of MRI, and this, in turn, made the surgical correction easier [60]. Therefore, MRI information can be used to improve modeling of prosthesis component that interacts with soft tissues and residual limb structures such as a socket and/or liner. However, similar to a CT scan, proper considerations regarding scanning parameters and scanner reconstruction algorithms are required to produce accurate 3D models of canine limb bones and soft-tissue structures using this method.

## Additive Manufacturing

Additive and subtractive manufacturing are commonly known as rapid prototyping. Additive manufacturing, typically referred to as “3D printing,” is currently the most used approach [62]. The American Society for Testing and Materials categorizes 3D printing approaches into vat polymerization, material extrusion, material jetting, binder jetting, powder bed fusion, sheet lamination, and directed energy deposition [62]. The 3D printer’s spectrum is wide due to all possible approaches, various materials, and 3D printer producers. However, primary considerations for prosthesis development may be the printing resolution, type of material, build size limit, and the use and removal of support structures. The unique advantages of developing a prosthesis with the use of a 3D printer include relatively reduced cost and the prosthesis can be patient-specific customized. Furthermore, compared to traditional prostheses, fabrication time and comfort of 3D printed prostheses can be reduced and improved, respectively [63]. Bachman *et al*. [15] presented an overview of the current market of prosthetics for amputated canines. The authors concluded that a customizable solution is the best option due to a great variation in canine limb shapes and dimensions [15]. Therefore, additive manufacturing approaches may be the correct alternative to produce patient-specific customized prosthesis. However, applications such as veterinary orthotics and prosthetics are not fully developed, which is evidenced by few studies reporting canine limb prosthesis cases [10,14,16,18,20,55,56].

### 3D-printed metallic prosthesis

The development of 3D-printed metallic prosthesis designs is intended to expedite osseointegration, reduce the operation and rehabilitation time, and to avoid the appearance of infections and pathology. At present, two primary approaches are utilized for metal powder bed additive manufacturing using laser for sintering or electron-beam for melting [17,64]. Indeed, common examples of such approaches are electro-beam melting (EBM) and selective laser sintering (SLS) [17]. In the EBM approach, heat produced by an electron beam fuses together the metallic powder positioned in a vacuum chamber. This approach differs from SLS as the metallic powder fuses having completely melted [65]. A previous study on additive manufacturing used in veterinary medicine reported that mechanical properties of EBM components manufactured from Ti-6Al-4V were better than the ones using SLS [10]. The conventional material for powder bed additive manufacturing of bone medical implants is Ti-6Al-4V powder, and it has been implemented in canine limb prosthesis fabrication in various studies [10,18,20].

## Biomechanical Analysis

Biomechanics analyzes and explains how muscles, bones, tendons, ligaments, and all elements of a living body work together to produce movement. For instance, an indirect parameter to quantify total limb function can be obtained by measuring ground reaction forces. In a canine, this measure indicates weight-bearing in the measured limb during gait [66,67]. A combination of kinetics with musculoskeletal geometry makes it possible to estimate joint and soft-tissue forces within the limb [67]. Research also combines 3D motion measurements and computational models to investigate muscle activation. Brown *et al*. [68] used the OpenSim modeling platform to develop a bilateral pelvic limb subject-specific rigid body musculoskeletal computer model of a canine. Muscle activation patterns, muscle forces, and angular kinematics and joint moments during walking were estimated by the model. Furthermore, the differentiation of normal and abnormal gaits is possible with proper quantification of canine gait analysis approaches [67]. For example, a previous study reporting on kinetic and kinematic analyses of thoracic or pelvic limb amputated canines indicated significant locomotive discrepancies when compared to quadrupedal canines [66]. Detrimental consequences on long-term musculoskeletal health and other quality of life problems can be caused by significant gait discrepancies [69]. Therefore, proper canine limb prosthesis design requires the inclusion of biomechanical analyses to optimize and evaluate prosthesis function.

### Motion capture technologies and equipment

Biomechanics has been applied to studying canine locomotion. Such research utilizes modern motion capture technologies and equipment to study canine 3D motion. These novel gait analysis technologies have also become available to assist veterinarians to diagnose numerous musculoskeletal and neurological conditions. A gait evaluation can greatly assist the implementation of correct orthopedic treatments and monitor their progression [70]. Skin marker motion analysis systems have been used in most of the former studies reporting on *in vivo* 3D kinematics of canines [66,67]. Although such systems allow for integration and synchronization of other devices, such as force plates and electromyography sensors, the kinematic data obtained from a skin marker-based motion capture system may be sensitive to soft tissue artifacts [71,72]. Cutting-edge imaging technology, such as the dual fluoroscopy imaging system, allows researchers to measure *in vivo* kinematics without being affected by soft-tissue artifacts [73,74]. Few studies have implemented this technology to investigate canine kinematics during several activities [75-77].

Canine movement analysis has made significant progress in imaging and video technology [67]. While marker motion analysis is still the most commonly used technology, future advancements in the dual fluoroscopy imaging technology will allow integration with multiple measuring systems to become more efficient and effective in research and development of highly functional canine limb prosthesis [77].

## Future Directions

Cutting-edge prosthetics innovation creates a conundrum when attempting to select what the best prosthetics is for a specific canine patient. The most optimal prosthesis for a canine is not determined by typical indicators such as materials, time of fabrication, complexity of design, ease of adaption, and cost [16,78]. The solution for replicating a normal limb function is to accommodate the features of the appropriate prosthetic with functional capabilities of the amputated canine. Matching the features of the proper prosthetic with the correct functionality is one of the main goals sought by the rehabilitation team, as claimed by the majority of veterinarians. Yet, such an objective is not simple to obtain.

**Figure-4:**
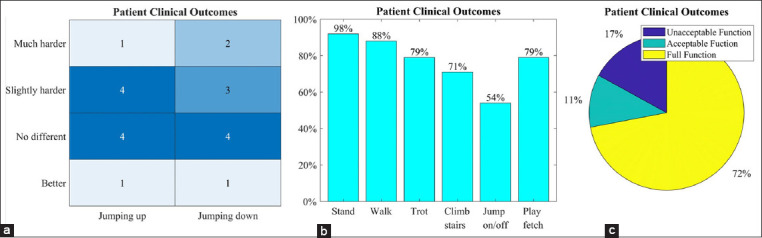
(a) The change in the ability to jump up and down compared to before fitting of the stump socket prosthesis (n=12) [79]. (b) Daily acts of living for patients after prosthetic placement, obtained from client answered surveys. Of the 24 patients, 91.66% (n=22) were able to stand using the prosthesis; 87.5% (n=21) were able to walk using the prosthesis; 79.17% (n=19) were able to trot using the prosthesis; 70.83% (n=17) were able to climb stairs using the prosthesis; 54.17% (n=13) were able to jump on or off furniture using the prosthesis; 79.17% (n=19) were able to play fetch using the prosthesis [16]. (c) Percentage of patient clinical outcomes for each range (n=47). Full function defined as restoration to, or maintenance of, full intended level and duration of activities and performance from pre-injury or pre-disease status (≥ 23 points). An acceptable function defined as restoration to, or maintenance of, intended activities and performance from pre-injury or pre-disease status that is limited in level or duration (12-22 points). The unacceptable function was defined as all other outcomes (≤ 11 points) with a possible score range of−13-34 [78].

As detrimental consequences on long-term musculoskeletal health and other quality of life problems can be caused by significant gait discrepancies [69], it is important to know the performance of canine limb prosthesis. To the best of our knowledge, up to now, outcomes of socket prosthesis placement in canines have been reported by only three retrospective studies [16,78,79] (Figure-4). One owner survey based investigation indicated that 83.3% of owners (10/12) communicated a good to excellent quality of life following prosthesis placement [79]. Another investigation indicated that 87.5% of patients (21/24) had the same to improved quality of life as they did prior to receipt of a prosthesis [16]. A third work reported that 89.3% of owners (42/47) said to have accepted to full function following prosthesis placement [78]. Although owner survey-based instruments can be used to evaluate owner perceptions of a canine quality of life and owner expectations regarding canine mobility, no investigation has been conducted on overall owner satisfaction with prostheses as a treatment option [78]. There is, then, a need for instruments to determine overall owner satisfaction with prostheses as a treatment option, gait alterations, as well as to evaluate the highest level of physical performance of canines with limb loss [16]. Thus, such indicators will objectively guide veterinarians to successfully accommodate the features of the appropriate prosthetic with functional capabilities of the amputated canine.

In addition to better instruments or indicators, more research needs to be conducted across multiple disciplines to enhance functionality of the residuum and canine-limb prostheses. For example, veterinarians and engineers should continue working together to improve the soft-tissue interface for osseointegration and cell adhesion to the titanium implant in order to prevent bacterial contamination, causing loosening, and subsequent bone loss [19,51]. Measuring, computational modeling, and finite element analysis research could be conducted on the socket-residual limb to better understand bone, skin, soft tissues, and load distribution interactions not only to obtain well-fitted and comfortable prostheses but also to eliminate sores, bruises, and other complications. Similar to humans, one approach could be to indirectly measure the kinematic interactions between the socket and the underlying bone using marker-based motion capture technology based on assumptions about joint constraints imposed on the system [80]. Further analysis involves the development and validation of tissue mechanics models using the collected marker-based information [81]. Moreover, most prostheses do not reproduce concentric muscle action and mainly have a passive function. Thus, the inclusion of muscle function needs to be explored to eventually improve the functionality of canine limb prostheses. One new device in human prosthesis traces muscle movement instead of muscle activation [82]. As an alternative to the conventional tracking myoelectrical signals, this method uses sensors to track the movement of implanted magnetic markers during muscle contraction. In human medicine, recent advances in prosthetic technology have enabled patients to recreate motor control and proprioception of the extremities in a way that restores the level of function they had before injury [46]. However, although some of these technological advances have been successfully applied in veterinary medicine [19], the development of multifunctional devices whose function replicates a healthy limb is far from complete in animals. For example, commercially available veterinary limb prostheses are limited in geometries and sizes, creating adaptation and functionality difficulties for patients, and possibly increasing the risk of prosthesis failure [18]. Furthermore, the number of kinematic studies in veterinary medicine is rising, yet unlike kinetic studies, there are no established protocols on how to collect kinematic data in canines [67].

## Conclusion

Considerable amounts of research and funding have been invested in the development of canine limb prosthesis designs. The main objective of these emerging technologies is to produce durable and highly functional canine limb prostheses. However, isolated research efforts toward the development of canine limb prosthesis may not be enough. Continued multidisciplinary research collaboration and teamwork among veterinarians, engineers, designers, and industry, with supporting scientific evidence, is required to better understand the development of canine limb prosthesis designs that closely replicate or mimic the normal limb function and will continue to be crucial in future research.

## Authors’ Contributions

PA and PC: Conception and design of the study, critical revision for important intellectual content, data acquisition, analysis, interpretation, and manuscript drafting. MG: Data acquisition, analysis, interpretation, and manuscript revision. IK and EAD: Conception and design of the study and critical revision for important intellectual content. All authors read and approved the final manuscript.
